# Effects of Polyphenol, Measured by a Biomarker of Total Polyphenols in Urine, on Cardiovascular Risk Factors After a Long-Term Follow-Up in the PREDIMED Study

**DOI:** 10.1155/2016/2572606

**Published:** 2016-01-04

**Authors:** Xiaohui Guo, Anna Tresserra-Rimbau, Ramón Estruch, Miguel A. Martínez-González, Alexander Medina-Remón, Olga Castañer, Dolores Corella, Jordi Salas-Salvadó, Rosa M. Lamuela-Raventós

**Affiliations:** ^1^Departament of Nutrition and Food Science, XaRTA, INSA, School of Pharmacy, University of Barcelona, Barcelona, Spain; ^2^Biomedical Research Networking Center-Physiopathology of Obesity and Nutrition (CIBEROBN), Carlos III Health Institute (ISCIII), Government of Spain, Madrid, Spain; ^3^Department of Internal Medicine, Hospital Clínic, IDIBAPS, University of Barcelona, Barcelona, Spain; ^4^Department of Preventive Medicine and Public Health, School of Medicine, University of Navarra, Pamplona, Spain; ^5^IdiSNA, Navarra Institute for Health Research, Pamplona, Spain; ^6^Cardiovascular Risk and Nutrition Research Group (CARIN, Regicor Study Group), Hospital del Mar Medical Research Institute (IMIM), Barcelona, Spain; ^7^Department of Epidemiology, Preventive Medicine and Public Health, School of Medicine, University of Valencia, Valencia, Spain; ^8^Human Nutrition Unit, University Hospital of Sant Joan de Reus, Department of Biochemistry and Biotechnology, Faculty of Medicine and Health Sciences, IISPV, Rovira i Virgili University, Reus, Spain

## Abstract

Several epidemiological studies have shown an inverse association between the consumption of polyphenol-rich foods and risk of cardiovascular diseases. However, accuracy and reliability of these studies may be increased using urinary total polyphenol excretion (TPE) as a biomarker for total polyphenol intake. Our aim was to assess if antioxidant activity, measured by a Folin-Ciocalteu assay in urine, is correlated with an improvement in cardiovascular risk factors (blood pressure and serum glucose, cholesterol, HDL-cholesterol, LDL-cholesterol, and triglyceride concentrations) in an elderly population at high risk. A longitudinal study was performed with 573 participants (aged 67.3 ± 5.9) from the PREDIMED study (ISRCTN35739639). We used Folin-Ciocalteu method to determine TPE in urine samples, assisting with solid phase extraction. Participants were categorized into three groups according to changes in TPE. Multiple linear regression models were used to assess relationships between TPE and clinical cardiovascular risk factors, adjusting for potential confounders. After a 5-year follow-up, significant inverse correlations were observed between changes in TPE and plasma triglyceride concentration (*β* = −8.563; *P* = 0.007), glucose concentration (*β* = −4.164; *P* = 0.036), and diastolic blood pressure (*β* = −1.316; *P* = 0.013). Our results suggest that the consumption of more polyphenols, measured as TPE in urine, could exert a protective effect against some cardiovascular risk factors.

## 1. Introduction

Cardiovascular diseases (CVDs) are considered to be the leading global cause of death, accounting for 17.3 million deaths per year, which is predicted to rise to more than 23.6 million by 2030 [1]. The main causes of CVDs involve nonmodifiable risk factors, such as age, sex, and family history of coronary heart disease (CHD), and modifiable risk factors, such as an unhealthy diet, lack of physical activity, smoking, and excessive alcohol intake [2, 3]. Therefore, an improvement of dietary habits could help to prevent CVDs.

Several studies have described protective roles of polyphenols in the cardiovascular system. The cardiovascular protection by polyphenol consumption can be explained by various mechanisms, including their anti-inflammatory properties, antioxidant capacity, improvement in endothelial function, inhibition of platelet aggregation and antithrombotic properties, and mechanisms that are not mutually exclusive [4–8]. Hence, further exploration of polyphenol consumption will help to discern its beneficial effects on human health. Prior information on polyphenol intake has often been collected through food frequency questionnaires (FFQs) or dietary recalls, whose bias can result in data not so accurate [9]. Therefore, in order to analyse associations between polyphenol intake and main cardiovascular risk factors, there is a need for biomarkers that can accurately reflect polyphenol intakein human studies.

The Folin-Ciocalteu method, an antioxidant assay based on electron transfer that measures the reductive capacity of an antioxidant, has been widely applied for measuring total polyphenol content in plant-derived food and recently in biological samples for clinical studies [10, 11]. Briefly, polyphenols from urine samples react with the Folin-Ciocalteu reagent to form a blue complex in alkaline medium, measured in spectrophotometry at 765 nm [12]. A solid phase extraction method is used to clean up the sample from possible interferences. This measurement of total urinary polyphenol excretion (TPE) has been considered as reliable biomarker of total polyphenol intake in recent years [8, 13, 14].

Several studies have addressed the relationship between polyphenol intake and cardiovascular risk factors; however, the results have led to mixed and inconsistent conclusions. Two studies conducted in healthy participants observed that improvement in cardiovascular health was due to higher HDL levels after intake of polyphenol-rich foods [15, 16]. Different results were obtained in other two studies in overweight subjects: one showed cardioprotective effects due to a reduction in body weight and an improvement in total cholesterol and LDL concentration after ingestion of a polyphenol extract from* Ecklonia cava*, while the other study observed a reduction in fasting glucose concentration when supplied with polyphenol-rich dark chocolate [17, 18]. Additionally, reduction in systolic blood pressure was observed in hemodialysis patients after the consumption of a polyphenol-rich beverage for one year [19]. Moreover, in the frame of the PREDIMED study, we found that specific categories of polyphenols, calculated through yearly FFQs and the Phenol-Explorer database, were significantly associated with decreased CVD risk [20].

Most of the aforementioned studies were conducted in small populations or over short periods of time. The association between polyphenol intake and cardiovascular risk factors has also been evaluated in large, long-term epidemiological trials, but with the limitations associated with using FFQs [21–24]. Therefore, the aim of the present study was to apply the reliable and validated antioxidant activity test, the Folin-Ciocalteu method, in urine samples as a biomarker of total polyphenol intake, to analyse the association between polyphenol intake and cardiovascular risk factors in an elderly population at high cardiovascular risk after a long-term follow-up (median: 4.8 years).

## 2. Methods

The present study was conducted within the frame of the PREDIMED study, which aimed to assess effects of the Mediterranean diet on the primary prevention of CVDs in Spain. The protocol and recruitment methods have been reported in detail elsewhere [25]. Eligible participants were men aged 55–80 and women aged 60–80 years without any history of cardiovascular disease but fulfilling at least one of the following two criteria: type-2 diabetes or three or more cardiovascular risk factors (family history of early-onset CVDs, hypertension, current smoking, low HDL-cholesterol, high LDL-cholesterol, and overweight or obesity). Exclusion criteria included any severe chronic illness, previous history of CVDs, alcohol or drug abuse, body mass index (BMI) of more than 40 kg/m^2^, and history of allergy or intolerance to olive oil or nuts. The trial was stopped after a median follow-up of 4.8 years due to the benefit of the Mediterranean diet with respect to major cardiovascular events: myocardial infarction, stroke, or death from cardiovascular causes (analysis performed by the Drug and Safety Monitoring Board of the trial), compared to a control low-fat diet [26].

The present longitudinal analysis included 612 volunteers, randomly selected from two recruitment centers in Spain. All participants provided written informed consent, and the protocol was approved by the Institutional Review Boards of the participating centers and registered.

### 2.1. Nutritional Assessments

Dietary habits of participants were assessed through a validated 137-item FFQ [27]. Nutrient intake was adjusted by calories using the residuals' method. Information about lifestyle, health condition, education, history of illnesses, and medication use was collected by a 47-item general questionnaire. The degree of adherence to the Mediterranean diet was assessed by a 14-point questionnaire [28]. Physical activity was assessed using the validated Spanish version of the Minnesota Leisure-Time Physical Activity Questionnaire [29]. All questionnaires were administered and repeated annually during the follow-up by trained staff in face-to-face interviews.

Information on polyphenol intake was obtained using the FFQ and the Phenol-Explorer database. The relationship between food items in the FFQ and the database has been described previously [30]. The content of total polyphenol intake equals the sum of all the individual polyphenol from each food item.

### 2.2. TPE Measurements

Urine samples were collected and coded and then immediately shipped to a central laboratory, to be stored at −80°C until analysed. The Folin-Ciocalteu method was applied to determine the content of TPE, using a clean-up procedure with solid phase extraction (SPE) performed in 96-well plate cartridges (Oasis MAX), which helped to remove urinary interferences. Finally, TPE was expressed as mg gallic acid equivalent (GAE)/g of creatinine. All details have been previously described by Medina-Remón et al. [14].

### 2.3. Clinical Measurements

Weight and height were measured with light clothing and no shoes with a calibrated balance and a wall-mounted calibrated stadiometer, respectively. BMI was calculated as weight in kilograms divided by the square of height in meters. For the measurement of blood pressure (BP), a validated semiautomatic sphygmomanometer (Omron HEM-705CP) was used by trained nurses. Measurements were taken at 5-minute intervals with participants in a seated position. Data were collected as an average of 2 measurements in each arm, repeated twice [31].

Plasma glucose, total cholesterol, and triglyceride concentrations were measured using standard enzymatic automated methods. Levels of HDL-cholesterol were measured by an enzymatic procedure after precipitation, and LDL-cholesterol was estimated by the Friedewald formula [32].

### 2.4. Statistical Analysis

Results were expressed as mean ± SD for continuous variables or percentages for categorical variables. Kolmogorov tests were applied to examine the normality distribution and skewness. All participants were divided into three categories according to changes in TPE during the follow-up (ΔTP < −11.4 mg gallic acid/g creatinine, −11.4 ≤ ΔTP ≤ 24.6 mg gallic acid/g creatinine, and ΔTP > 24.6 mg gallic acid/g creatinine). Changes in nutrient and key food consumption during the follow-up were assessed with ANOVA for repeated measurements analysis. Bonferroni* post hoc* test and paired* t*-test were used to compare each variable within and between groups.

Multivariate linear regression models were used to assess the relationship between serum glucose, total cholesterol, HDL, LDL, triglyceride concentrations, systolic blood pressure (SBP), diastolic blood pressure (DBP), and heart rate, and tertiles of changes in TPE during the follow-up period, adjusted for potential confounders (sex, age, intervention groups, BMI, smoking status, family history of CHD, physical activity, hypertension, diabetes, dyslipidemia, medication use, and 14-unit Mediterranean diet score at baseline). Sensitivity analyses were used to further assess the relationship between specific cardiovascular risk factors and subcategories.

General Linear Model (GLM) approach to ANCOVA was used to determine differences between tertiles of changes in TPE after 5-year follow-up, adjusted for potential confounders as did in multivariate linear regression models.

All analyses were performed using SPSS software V21.0 (Chicago, USA). All models were tested for the detection of outliers, multicollinearity, homoscedasticity, and normality and independence of errors. All statistical tests were two-tailed, and the significance level was *P* < 0.05.

## 3. Results

After 5 years of follow-up of 612 participants randomly selected for this substudy of the PREDIMED trial, 39 were excluded because of extreme TPE values, hence a total of 573 participants were included in the present study. Baseline characteristics of participants grouped by tertiles of changes in TPE during the follow-up are shown in Table 1. According to the study design, the average age was 67.3 ± 5.9 years with a BMI of 29.2 ± 3.3 kg/m^2^. Most of the participants gathered a high number of cardiovascular risk factors: 41.5% had diabetes; 80.5% had hypertension; 66.8% had dyslipidemia; 16.9% were current smokers, and 37.5% had a family history of CHD. In the second tertile, individuals were less likely to be women and had a higher body weight.

Table 2 shows changes in key food consumption during the follow-up. Most key foods changed considerably after the long-term intervention, with the exception of legumes and chocolate.  Table 3 summarizes information on nutrient intake at baseline and 5 years according to changes in TPE during the follow-up. Comparing nutrient intake at 5 years versus baseline, we observed a significant increase in total fat, fibre, monounsaturated fatty acids (MUFA), polyunsaturated fatty acids (PUFA), K, and Mg, while other items such as total carbohydrates, protein, saturated fatty acids (SFA), Na, and cholesterol remained unchanged. This may be due to dietary changes based on recommendations to adhere to a Mediterranean diet, which is characterized by a high consumption of vegetables, fruits, olive oil, wine, and nuts and a low consumption of red meat, high-fat dairy products, and sweets. However, there were no significant changes when comparing tertiles and their interaction. In addition, we found that significant changes in TPE did not significantly affect the intake of nutritional elements among groups.

Several antioxidant substances such as sulfur dioxide, ascorbic acid, sugar, aromatic amines, organic acid, Fe(II), and nonphenolic organic substances might affect total polyphenol when applying Folin-Ciocalteu assay; however, after a solid phase extraction (SPE), the aforementioned interfering substances were eliminated through the cleaning-up process [14].

Linear regression analyses were conducted to assess the relationship between TPE (*Q*_1_ = −48.70 ± 30.11 mg GAE/g creatinine;* Q*_2_ = 6.95 ± 10.55 mg GAE/g creatinine;* Q*_3_ = 64.48 ± 31.61 mg GAE/g creatinine) and clinical possible cardiovascular risk factors (plasma glucose, triglyceride, cholesterol, HDL-c, and LDL-c concentrations, and SBP, DBP, and heart rate). Results are shown in Table 4. Significant inverse associations were found between tertiles of changes in TPE and glucose (*β* = −4.372; *P* = 0.026), triglycerides (*β* = −8.572; *P* = 0.006), and DBP (*β* = −1.156; *P* = 0.031) after adjustment for potential confounders. However, other parameters did not show significant associations. The standardized coefficients (Beta) in the model were used to measure degrees of contribution to different risk factors. Results indicate that, among the CVD risk factors, triglyceride levels showed the highest beneficial effects of dietary polyphenol intake (Beta = −0.126; *P* = 0.031).

We also conducted sensitivity analyses to ascertain whether significant changes were related to specific variables. As shown in Table 5, men were more likely to improve their plasma triglyceride concentration than women, according to tertiles of changes in TPE. In contrast, the lowering effects of higher polyphenol consumption on DBP were greater in women. In addition, when the P-14 was considered separately, higher scoring groups showed significant differences in plasma triglyceride concentration according to tertiles of changes in TPE.

## 4. Discussion

In this 5-year study of an elderly population at high cardiovascular risk living in a Mediterranean country, we observed that higher polyphenol intake, measured by TPE, was inversely associated with some cardiovascular risk factors. The observed benefits on CVDs were ascribable to a reduction in plasma glucose and triglyceride concentrations and a diminution of DBP. This may partly explain the decreased CVD risk shown by people following a polyphenol-rich diet such as the Mediterranean diet.

The beneficial effects of polyphenols consumption on major cardiovascular events in the PREDIMED cohort have been published before [20]. The difference between our findings and other reported results lies in the measurement of polyphenols in urine as biomarker of polyphenol intake. Given that more than 8000 phenolic structures exist in nature, beneficial effects from polyphenols depend on a variety of factors, including total intake, food cooking processes, digestion, absorption, metabolic pathways* in vivo*, or even differences between individuals [33]. Therefore, TPE, as a biomarker of total polyphenol intake, may provide a more accurate insight into the effects of polyphenols on CVDs than other dietary assessment methods.

Previous clinical studies on the benefits of polyphenols on the cardiovascular system have provided inconsistent results. A 12-week follow-up clinical trial conducted in Korea reported a strong inverse association between consumption of polyphenol extracts from* Ecklonia cava* and serum glucose, SBP, and HDL concentration [17]. In contrast, a recent randomized control trial performed with 67 elderly men at high cardiovascular risk found an increase in HDL after consumption of red wine, whereas fasting glucose was kept constant throughout the study, which differs from our observation [34]. Another contrasting result was found in participants with type-2 diabetes, who improved their HDL level and decreased total cholesterol after the consumption of polyphenol-rich chocolate [35]. In addition, a group of overweight participants consuming polyphenol-rich dark chocolate had lower plasma glucose, SBP, and DBP after the intervention, which partly agrees with our findings [18]. However, in the present study, we found no association between polyphenol intake and cholesterol profiles or SBP.

Participants who increased their polyphenol intake showed a reduction in plasma glucose concentrations, adding to the evidence that polyphenol-rich diets protect the cardiovascular system by improvements in glycemic control. A similar clinical trial performed on 78 participants at high cardiovascular risk, administration of polyphenol-rich foods, improved glucose metabolism by increasing early insulin secretion and insulin sensitivity [36]. Another cross-sectional study in an elderly population reported that green tea consumption was inversely associated with fasting blood glucose concentrations, though without adjusting for potential confounders [37]. Despite the abundance of results from different clinical trials, animal models, and* in vitro* tests, the mechanisms for hypoglycemic effects of polyphenols still warrant discussion. Potential explanations for these putative protective effects include reduced absorption of total carbohydrate in the intestine, modulation of enzymes related to glucose metabolism, stimulation of insulin secretion, improvement of *β*-cell function and insulin action, reduction in oxidative stress, inhibition of glucose transport, and enhanced vascular function [36, 38–40].

Triglycerides are considered the highest source of energy, and inhibition of triglyceride absorption also plays a role in the prevention of CVDs [41]. In the present study, increasing polyphenol intake was inversely associated with triglyceride levels, in agreement with some previous studies. Sugiyama et al. investigated the inhibitory effect of oligomeric procyanidins from apples on triglyceride absorption, explained by the inhibition of pancreatic lipase activity* in vivo* and in animal models [42]. Data from animal models indicated that such lowering effects could be attributed to the very low-density lipoprotein (VLDL) secretion rates and a decrease in apolipoprotein B secretion [43]. In addition, a study of haemodialysis patients fed with polyphenol-rich pomegranate juice also reported improvements in triglyceride levels, but this was explained by an inhibition of intestinal absorption and clearance of plasma triglycerides* in vivo*[19]. The variety of plausible mechanisms put forward to explain these effects, such as absorption, metabolism, and elimination during metabolic processes, reflect the highly varied chemical structure of polyphenols. Unlike the current study, most clinical trials have focused on a single polyphenol-rich food such as dark chocolate, wine, or green tea. Therefore, considering that the Mediterranean diet is a constellation of several polyphenol-rich foods, it is difficult to draw a single mechanism to explain the lowering effects found on triglycerides.

Hypertension is a well-established risk factor for CVDs [44]. There is evidence from our study and others that increasing polyphenol intake is associated with lower BP. Both DASH (Dietary Approaches to Stop Hypertension) and SUN (Seguimiento Universidad de Navarra) studies emphasize that the consumption of plant-derived foods, particularly fruits, vegetables, nuts, and olive oil, is inversely associated with BP [45–47]. We previously reported that greater TPE was inversely associated with BP [13]. However, we found significant associations only for DBP, and not SBP. Another PREDIMED clinical substudy based on a 4-year intervention also supports our findings [48]. Mechanisms of the BP lowering effect could involve endothelial nitric oxide (NO) production. NO plays a fundamental role in the regulation of the vascular system, and vascular homeostasis is achieved only when NO levels are adequate [6]. Briefly, polyphenols induce NO production by promoting endothelial nitric oxide synthase (eNOS) expression, generating vascular relaxing factors such as prostacyclin (PGI2) and inhibiting synthesis of the vasoconstrictor endothelin-1 (ET-1) in vascular endothelial cells [49]. Strong and positive association between polyphenol intake and plasma NO levels has been previously demonstrated by our group [8].

Some study limitations deserve to be noted. First, given that this substudy was conducted only among elderly subjects at high cardiovascular risk, it is difficult to extrapolate the results to the general population. Second, even though we adjusted potential confounders relative to CVD risk, residual confounding could still exist. Nonetheless, our study adds new evidence in support of a preventative effect of a long-term polyphenol intake on CVDs.

Compared with previous studies, the present study also has several strengths. Firstly, even though biomarkers are necessary to assess the compliance of the intervention, it is difficult to find a reliable and available biomarker. TPE in urine could be useful as a marker of compliance in intervention studies with foods with high-polyphenol content such as fruits, vegetables, wine, chocolate, tea, and coffee, while other markers are not suitable; moreover, in comparison with measuring the total polyphenol intake through self-reported information based on FFQ, the use of TPE, a biomarker of polyphenol intake, could provide more precise evidence [20, 50]. Secondly, the long duration of the intervention should also be considered as strength, since only few studies have tested associations between polyphenols and cardiovascular risk factors in such long-term intervention [8, 51, 52]. Thirdly, the selection of participants is a group of free-living individuals reproducing real-life conditions with home-prepared, energy-unrestricted foods. Fourthly, the Folin-Ciocalteu assay is a rapid, cheaper, and environmentally friendly measurement without requirement of dedicated instrumentation, which could be suggested to be applied in large intervention studies in the future.

In conclusion, in this 5-year study within the frame of the PREDIMED trial conducted in subjects at high cardiovascular risk, we found that polyphenol intake measured by TPE was inversely associated with some clinical cardiovascular risk factors, namely, plasma glucose and triglycerides concentrations and SBP, suggesting that intake of polyphenols provides protection against CVDs throughout these mechanisms. Further research is needed to confirm the current findings in the general population.

## Figures and Tables

**Table 1:** Baseline characteristics of participants according to tertiles of changes in TPE.

	TPE (mg GAE/g creatinine)						
	*Q*_1_		*Q*_2_		*Q*_3_		*P*
	(ΔTP < −11.4)		(−11.4 ≤ ΔTP ≤ 24.6)		(ΔTP > 24.6)		
Number of subjects	191		191		191		
Women, *n* (%)	101	(52.9)	83	(43.5)	112	(58.6)	0.011
Age (y), mean (SD)	66.7	(5.9)	67.3	(5.8)	68.00	(6.0)	0.113
Weight (kg), mean (SD)	73.9	(10.6)	77.1	(11.6)	74.5	(10.7)	0.01
BMI (kg/m^2^), mean (SD)	28.9	(3.1)	29.6	(3.5)	29.2	(3.2)	0.103
Systolic BP (mm Hg), mean (SD)	149.8	(17.9)	151.6	(16.9)	152.8	(18.6)	0.238
Diastolic BP (mm Hg), mean (SD)	84.3	(9.8)	85.9	(10.0)	85.5	(10.4)	0.269
Hypertension, *n* (%)	151	(79.1)	152	(79.6)	158	(82.7)	0.621
Diabetes, *n* (%)	78	(40.8)	85	(44.5)	75	(39.3)	0.567
Dyslipidemia, *n* (%)	136	(72.3)	117	(61.3)	128	(67)	0.074
Smoking status							0.641
Current, *n* (%)	35	(18.3)	34	(17.8)	28	(14.7)	0.586
Former, *n* (%)	36	(18.8)	43	(22.5)	47	(24.6)	0.388
Never, *n* (%)	120	(62.8)	114	(59.7)	116	(60.7)	0.814
Family history of CHD, *n* (%)	65	(35.3)	75	(40.3)	75	(41.2)	0.460
Medication							
Aspirin, *n* (%)	33	(32.0)	35	(34.0)	35	(34.0)	0.949
Antihypertensive drugs, *n* (%)	131	(68.6)	142	(74.3)	141	(73.8)	0.381
Hypolipidemic drugs, *n* (%)	91	(47.6)	70	(36.6)	78	(40.8)	0.089
Insulin, *n* (%)	10	(5.2)	9	(4.7)	8	(4.2)	0.890
Oral hypoglycemic drugs, *n* (%)	40	(20.9)	46	(24.1)	45	(23.6)	0.736
Vitamin or minerals, *n* (%)	18	(9.5)	16	(8.5)	13	(6.9)	0.644
Educational level							
Primary school, *n* (%)	140	(74.1)	139	(73.5)	146	(76.8)	0.793
High school, *n* (%)	32	(16.9)	28	(14.8)	28	(14.7)	
University, *n* (%)	17	(9.2)	22	(11.6)	16	(8.4)	
Physical activity at leisure time (MET-min/d)	275	(212)	287	(204)	269	(183)	0.696
Polyphenol intake (mg/d)	853.4	(239.8)	831.2	(248.9)	882.7	(247.8)	0.135

BMI: body mass index; CHD: coronary heart disease; GAE: gallic acid equivalent; TPE: total polyphenol excretion.

Data are given as means (SD) for continuous variables and percentages for categorical variables; *P* < 0.05 indicates statistical significance.

^*∗*^ *P* values calculated by analysis of variance or *χ* ^2^ tests.

**Table 2:** Changes in daily intake of key foods after 5 years with energy adjustment categorized by tertile of changes in TPE^a^.

		TPE (mg GAE/g creatinine)								
		*Q*_1_		*Q*_2_		*Q*_3_		*P* ^b^		
		(ΔTP < −11.4 )		(−11.4 ≤ ΔTP ≤ 24.6)		(ΔTP > 24.6)				
		Mean	SD	Mean	SD	Mean	SD	Time^c^	Group^d^	Time *∗* Group^e^
Vegetables (g/d)	Baseline	302.1	117.5	293.9	109.0	289.6	118.4	<0.001	0.195	0.369
	5 years	366.0^*∗∗*^	122.9	340.7^*∗∗*^	115.8	354.3^*∗∗*^	120.9			
Fruits (g/d)	Baseline	346.3	176.7	354.7	169.3	385.4	183.6	<0.001	0.477	0.103
	5 years	459.4^*∗∗*^	181.4	456.4^*∗∗*^	172.6	454.0^*∗∗*^	158.8			
Legumes (g/d)	Baseline	18.7	7.2	19.2	7.2	19.69	8.8	0.445	0.149	0.736
	5 years	18.7	8.3	19.1	7.7	19.9	8.1			
Cereals (g/d)	Baseline	240.0	73.2	242.9	79.2	238.9	70.7	<0.001	0.867	0.712
	5 years	221.1^*∗∗*^	63.4	216.4^*∗∗*^	68.5	216.1^*∗∗*^	63.0			
Milk (g/d)	Baseline	368.8	201.3	345.4	193.9	393.2	233.2	0.005	0.300	0.166
	5 years	402.2^*∗*^	223.4	386.4^*∗∗*^	204.3	395.6	196.6			
Meat (g/d)	Baseline	140.9	49.1	140.1	48.8	138.0	45.3	<0.001	0.992	0.431
	5 years	126.6^*∗∗*^	41.9	126.9^*∗∗*^	43.3	130.1	44.1			
Fish (g/d)	Baseline	94.7	37.2	90.4	39.1	91.7	39.2	<0.001	0.521	0.882
	5 years	101.4	45.6	98.8^*∗∗*^	43.2	97.8^*∗*^	36.2			
Pastries (g/d)	Baseline	26.1	26.1	25.7	27.1	26.6	25.1	0.001	0.846	0.485
	5 years	20.1	24.2	23.1	28.4	21.5	27.3			
EVOO (g/d)	Baseline	24.1	24.2	21.8	23.8	21.3	22.9	<0.001	0.848	0.346
	5 years	48.2^*∗∗*^	22.8	48.1^*∗∗*^	25.0	49.7^*∗∗*^	23.1			
Nuts (g/d)	Baseline	11.0	12.1	9.8	13.1	10.6	13.1	<0.001	0.794	0.656
	5 years	16.0^*∗∗*^	12.5	16.1^*∗∗*^	13.1	16.7^*∗∗*^	12.2			
Wine (g/d)	Baseline	98.3	140.1	105.2	157.2	96.8	136.1	0.002	0.781	0.979
	5 years	80.1^*∗∗*^	130.5	89.0	130.8	80.7	123.4			
Folic acid (*μ*g/d)	Baseline	376.7	83.8	379.4	81.1	381.9	87.6	<0.001	0.939	0.322
	5 years	432.6^*∗∗*^	75.9	425.3^*∗∗*^	87.3	424.5^*∗∗*^	72.4			
Coffee (mL/d)	Baseline	38.4	57.0	33.2	44.2	33.9	47.2	0.004	0.902	0.258
	5 years	27.4^*∗*^	48.0	28.6	46.4	30.7	48.8			
Chocolate (g/d)	Baseline	2.9	5.7	2.5	4.7	3.1	5.9	0.940	0.422	0.203
	5 years	2.2	4.2	3.1^*∗*^	6.1	3.4	7.9			

^a^Data are given as means (SD); *P* < 0.05 indicates statistical significance. EVOO: extra virgin olive oil; GAE: gallic acid equivalent; TPE: total polyphenol excretion. Values with asterisks are statistically different from baseline values by the paired-samples *t*-test (^*∗*^ *P* < 0.05; ^*∗∗*^ *P* < 0.01).

^b^Data analysed by repeated-measures 2-factor ANOVA.

^c^Comparison between the time before and after intervention.

^d^Comparison between tertiles of TPE changes.

^e^Comparison between measurements obtained before and after intervention and between tertiles of TPE changes.

**Table 3:** Changes in nutrient intake after 5 years with energy adjustment categorized by tertile of changes in TPE^a^.

		TPE (mg GAE/g creatinine)								
		*Q*_1_		*Q*_2_		*Q*_3_		*P* ^b^		
		(ΔTP < −11.4)		(−11.4 ≤ ΔTP ≤ 24.6)		(ΔTP > 24.6)				
		Mean	SD	Mean	SD	Mean	SD	Time^c^	Group^d^	Time *∗* Group^e^
Total carbohydrates (g/d)	Baseline	235.6	36.5	238.4	43.2	239.9	35.9	0.736	0.964	0.41
	5 years	239.7	63.1	235.0	68.9	235.6	61.0			
Protein (g/d)	Baseline	88.4	36.5	91.2	43.2	92.7	35.9	0.12	0.649	0.498
	5 years	94.5	18.6	92.7	19.5	94.4	17.7			
Total fat (g/d)	Baseline	102.5	12.8	100.8	12.6	102.7	13.6	<0.001	0.528	0.331
	5 years	110.7^*∗∗*^	23.1	112.9^*∗∗*^	25.6	113.4^*∗∗*^	24.4			
MUFA (g/d)	Baseline	53.5	13.6	52.3	17.4	51.6	15.2	<0.001	0.97	0.19
	5 years	58.3^*∗∗*^	12.7	59.6^*∗∗*^	13.5	59.8^*∗∗*^	13.5			
SFA (g/d)	Baseline	25.5	9.0	24.6	10.1	24.0	9.7	0.949	0.887	0.114
	5 years	24.2	6.4	24.8	7.4	25.1	7.3			
PUFA (g/d)	Baseline	15.7	4.7	15.7	6.1	15.6	5.4	<0.001	0.716	0.675
	5 years	19.0^*∗∗*^	5.9	19.0^*∗∗*^	5.6	19.6^*∗∗*^	5.5			
Alcohol (g/d)	Baseline	14.1	5.2	13.3	5.4	13.4	4.8	0.039	0.979	0.75
	5 years	11.9	14.6	12.4	15.2	12.2	14.7			
Fibre (g/d)	Baseline	24.2	6.0	24.6	5.6	25.2	6.4	<0.001	0.564	0.204
	5 years	26.6^*∗∗*^	7.5	25.8	7.4	26.4	7.0			
Cholesterol (mg/d)	Baseline	352.4	84.6	353.1	94.6	350.5	94.0	0.2	0.946	0.975
	5 years	359.9	90.9	358.0	98.7	356.8	92.7			
Na (mg/d)	Baseline	2322.4	479.6	2273.1	528.7	2263.7	479.9	0.736	0.963	0.41
	5 years	2229.8	644.5	2230.8	728.0	2253.7	652.0			
K (mg/d)	Baseline	4230.9	723.7	4164.3	682.7	4300.6	796.1	<0.001	0.234	0.542
	5 years	4654.5^*∗∗*^	826.8	4546.0^*∗∗*^	963.7	4614.7^*∗∗*^	805.9			
Mg (mg/d)	Baseline	359.5	62.7	358.4	58.1	367.1	61.8	<0.001	0.432	0.365
	5 years	398.5^*∗∗*^	82.1	388.1^*∗∗*^	86.4	394.3^*∗∗*^	80.8			

SFA: saturated fatty acids, MUFA: monounsaturated fatty acids, PUFA: polyunsaturated fatty acids, GAE: gallic acid equivalent; TPE: total polyphenol excretion.

^a^Data are given as means (SD); *P* < 0.05 indicates statistical significance. Values with asterisks are statistically different from baseline by paired-samples *t*-test (^*∗*^ *P* < 0.05; ^*∗∗*^ *P* < 0.01).

^b^Data analysed by repeated measures 2-factor ANOVA.

^c^Comparison between the time before and after intervention.

^d^Comparison between tertile changes in TPE.

^e^Comparison between measures obtained before and after intervention and between tertiles of TPE changes.

**Table 4:** Multivariate linear regression analyses with changes in cardiovascular risk factors as dependent variables and tertiles of changes in TPE in spot urine samples (mg GAE/g creatinine) as exposure variables, adjusted for potential confounders.

		*β*	SE	Beta	Sig.	95% CI	
Change in GLU (mg/dL)	Model 1	−4.164	1.979	−0.095	0.036	−8.053	−0.275
	Model 2	−4.316	1.981	−0.098	0.030	−8.208	−0.424
	Model 3	−4.355	1.949	−0.099	0.026	−8.186	−0.525
	Model 4	−4.372	1.953	−0.099	0.026	−8.209	−0.534

Change in COL (mg/dL)	Model 1	−2.51	2.001	−0.057	0.210	−6.442	1.421
	Model 2	−2.236	2.011	−0.050	0.267	−6.187	1.715
	Model 3	−1.845	2.013	−0.042	0.360	−5.800	2.109
	Model 4	−1.802	2.015	−0.041	0.372	−5.762	2.157

Change in HDL (mg/dL)	Model 1	0.102	0.448	0.010	0.820	−0.778	0.982
	Model 2	0.135	0.448	0.014	0.763	−0.744	1.015
	Model 3	0.133	0.456	0.014	0.771	−0.764	1.030
	Model 4	0.174	0.454	0.018	0.701	−0.718	1.067

Change in LDL (mg/dL)	Model 1	−0.205	1.775	−0.005	0.908	−3.693	3.283
	Model 2	−0.039	1.784	−0.001	0.983	−3.545	3.467
	Model 3	0.448	1.783	0.012	0.802	−3.056	3.952
	Model 4	0.469	1.786	0.012	0.793	−3.041	3.979

Change in TG (mg/dL)	Model 1	−8.356	3.06	−0.123	0.007	−14.369	−2.344
	Model 2	−8.563	3.058	−0.126	0.005	−14.572	−2.554
	Model 3	−8.627	3.094	−0.127	0.006	−14.708	−2.546
	Model 4	−8.572	3.099	−0.126	0.006	−14.662	−2.483

Change in SBP (mm Hg)	Model 1	−1.367	0.994	−0.058	0.169	−3.319	0.585
	Model 2	−1.222	1.001	−0.052	0.222	−3.188	0.744
	Model 3	−1.127	1.003	−0.048	0.262	−3.098	0.843
	Model 4	−1.098	1.005	−0.046	0.275	−3.071	0.876

Change in DBP (mm Hg)	Model 1	−1.316	0.531	−0.104	0.013	−2.359	−0.273
	Model 2	−1.254	0.532	−0.099	0.019	−2.298	−0.209
	Model 3	−1.153	0.532	−0.091	0.031	−2.198	−0.108
	Model 4	−1.156	0.533	−0.091	0.031	−2.203	−0.109

Change in HR	Model 1	−0.002	0.555	0.000	0.997	−1.091	1.087
	Model 2	0.043	0.559	0.003	0.938	−1.055	1.142
	Model 3	−0.011	0.567	−0.001	0.985	−1.125	1.103
	Model 4	−0.074	0.565	−0.006	0.895	−1.184	1.035

GLU: glucose, COL: total cholesterol, HDL: high-density lipoprotein, LDL: Low-density lipoprotein, TG: triglycerides, SBP: systolic blood pressure, DBP: diastolic blood pressure, and HR: heart rate.

*β*: nonstandardized coefficient (regression line coefficient); SE: standard error; Beta: standardized coefficient; CI: confidence interval; *P*: two-sided test of significance.

Model 1: unadjusted; Model 2 adjusted for sex, age, and intervention groups; Model 3 adjusted as in Model 2 plus BMI, smoking status, family history of CHD, physical activity, hypertension, diabetes, dyslipidemia, and medication use: antihypertensive drugs, vitamins, insulin, oral hypoglycemic drugs, aspirin, or other antiplatelet drug; Model 4 was adjusted as in Model 3 plus 14-unit Mediterranean diet score.

**Table 5:** Sensitivity analysis of clinical cardiovascular risk factors.

			Change in TG (mg/dL)							Change in GLU (mg/dL)							Change in DBP (mm Hg)						
		*N*	*Q*_1_		*Q*_2_		*Q*_3_		*P* ^a^	*Q*_1_		*Q*_2_		*Q*_3_		*P* ^a^	*Q*_1_		*Q*_2_		*Q*_3_		*P* ^a^
			Mean	SD	Mean	SD	Mean	SD		Mean	SD	Mean	SD	Mean	SD		Mean	SD	Mean	SD	Mean	SD	
Gender	Male	236	6.41	43.58	−17.86	62.54	−14.80	44.68	0.007	2.35	38.03	−0.32	35.56	−6.12	35.99	0.36	−1.24	10.04	−2.43	9.73	−2.14	11.67	0.714
	Female	249	8.22	46.57	9.25^*∗∗*^	53.14	−5.01	68.69	0.194	10.31	38.68	9.23	30.44	1.51	35.04	0.193	−1.46	10.24	−3.67	10.75^*∗*^	−5.29	9.84	0.026
Age, years	≤67	242	8.71	47.80	−9.75	70.39	−8.77	62.27	0.089	7.08	41.13	1.56	30.86	−2.23	36.48	0.262	−0.64	9.70	−1.41	10.78	−2.77	11.73	0.396
	≥68	243	5.96	42.21	−2.06	46.74	−9.68	57.35	0.129	6.06	35.60	6.37	36.39	−1.36	34.91	0.285	−2.18	10.58	−4.50^*∗*^	9.36	−4.99	9.75	0.123
P-14	<9	274	−0.89	57.27	−5.27	58.57	−10.63	67.61	0.676	7.29	28.72	5.65	34.89	−3.68	42.90	0.178	−1.36	11.63	−2.39	9.87	−4.98	10.05	0.094
	≥9	211	12.07	35.90	−6.49	61.19	−8.33	53.71	0.007	6.18	43.25	2.69	32.89	−0.43	29.55	0.428	−1.35	9.20	−3.36	10.40	−3.27	11.17	0.24

P-14: 14-point Mediterranean diet score test; GLU: glucose; TG: triglycerides; DBP: diastolic blood pressure.

^a^ *P* value tested by ANOVA.

Values with asterisks are statistically different from the baseline by the paired-samples *t*-test (^*∗*^ *P* < 0.05; ^*∗∗*^ *P* < 0.01).
